# Investigation regarding the application of the titanium electrode for the water treatment plant in a steel manufacturing plant

**DOI:** 10.3389/fchem.2022.1065332

**Published:** 2022-12-20

**Authors:** Jovana Gradinac, Aleksandar Jovović

**Affiliations:** Faculty of Mechanical Engineering, University of Belgrade, Belgrade, Serbia

**Keywords:** electrocoagulation (EC), water purification, hardness removal, water control UET system, titanium

## Abstract

Hard water causes problems in the industry since the deposits inside pipes and equipment lead to lower plant efficiency and electricity costs. The growing demands for high-quality water necessitate the development of modern and cost-effective technologies for softening very hard water. One of these techniques is the electrocoagulation process (EC). This study aimed to examine the effectiveness of the electrocoagulation (EC) process for removing scale ions in water using titanium rod electrodes. The research was carried out on pilot electrodes. The results that were obtained have focused on showing the effectiveness and efficiency of the application of titanium electrodes for removing hardness from makeup and process water inside a closed system, utilizing a Universal Environmental Technologies system (UET system). The plant consisted of a heat pump, heat exchanger, cooling tower, and Universal Environmental Technologies reactor with a titanium rod.

## 1 Introduction

Recirculating cooling water systems are broadly applied to cool equipment and pipelines in the fields of petrochemical processing, steel manufacturing, and electricity generation (Hasson et al., 2008; Nishida et al., 2009; Zaslavschi et al., 2013).

Due to the evaporation effect, recirculating process cooling water usually contains a high concentration of ions, particularly magnesium (Mg^2+^) and calcium (Ca^2+^), which cause the water to become hard and form scales on the inner pipeline surfaces (Park et al., 2007).

In a few types of research, the efficiency of the application of titanium electrodes for removing the hardness from makeup and process water inside a closed system and whether the water inside the system, after being returned to a “natural” balance, would remain in the same state were studied (Flynn, 2009).

The control of scale deposits in cooling water systems is necessary to maintain maximum heat transfer efficiency. The formation of scales in the cooling tower can be controlled by controlling the cooling tower blowdown water to limit the cycles of concentration (Flynn, 2009). The volume of blowdown water produced during the operation could be reduced by removing or eliminating the scale-forming ions from cooling tower water and then recycling the clean water from the cooling tower circuit (Abdel-Shafy et al., 2020).

Compared with using fresh water, using recycled clean water can produce substantial gains because of the large volumes of water required in cooling towers (Herro and Port, 1993). There are several treatment technologies to reduce scaling in cooling water systems, such as reverse osmosis, nanofiltration, ion exchange, and chemical coagulation methods (Amjad and Demadis, 2015; Abdel-Shafy and Abdel-Shafy, 2017; Al-Sa’ed et al., 2011; Abdel-Shafy, 2015).

However, these technologies, due to the effect of fouling on the membranes used, are not suitable for treating cooling water with high levels of scale-forming ions (Abdel-Shafy and Abdel-Shafy, 2017). Electrochemical methods have been found effective to treat industrial water (Brillas et al., 2009; Nidheesh et al., 2013; Nidheesh et al., 2019). Electrochemical methods were, and still are, an understudy for those cases where the traditional methods are not efficiently enough to achieve the concentration limits imposed by law or when they appear more economically convenient (De Francesco and Costamagna, 2004).

Industrial water usually contains enough dissolved solids to enhance the efficiency of electrochemical processes such as electrocoagulation (Kabdas¸ lı et al., 2012; Gandhimathi et al., 2015), indirect electrochemical oxidation (Mohan et al., 2007; Rajkumar and Palanivelu, 2014), anodic oxidation (Panizza and Cerisola, 2006; Tsantaki et al., 2012), and electro-Fenton processes (Oturan et al., 2015; Roshini et al., 2017). These processes have received much attention due to their efficiency in treating water containing high levels of solids.

The electrochemical purification of water contaminated by pollutants generally dissolves simple and harmless molecules in a fluid (gas or more often liquid) through electrochemical reactions (De Francesco and Costamagna, 2004).

The most important reactant in electrochemical purification is the electron, a “clean reagent,” which explains the low waste output associated with electrochemical and electrocoagulation methods (Kuokkanen et al., 2013). One of the main advantages of the electrochemical and electrocoagulation processes is that no chemicals are used, so these methods are considered green technology (Atwood, 2016).

The process of electrocoagulation involves the formation of coagulants by creating metal ions on the anode reactor; destabilization of contaminants, suspension of particles, and breaking of the emulsion; aggregation of unstable phases; and flocculant formation by introducing an electrical current into the medium (De Francesco and Costamagna, 2004).

The anodic dissolution produces *in situ* coagulants (metallic ions) (Eq. 1) with the generation of hydroxyl ions (Eq. 3) and hydrogen gas at the cathode (Eq. 2). These *in situ* coagulants are responsible for the generation of flocs surrounded by metal hydroxides (Nidheesh and Singh, 2017), which act as an excellent adsorbent.

At the anode,

$$M_{\left(s\right)}\to M_{\left(aq\right)}^{n+}+ne^{-}$$ (1)

$$H_{2}O\to 4H_{\left(aq\right)}^{+}+O_{2\left(g\right)}+4e^{-}$$ (2)

At the cathode,

$$nH_{2}O+ne^{-}\to \left(\frac{n}{2}\right)H_{2\left(g\right)}+nOH^{-}$$ (3)

The basic principle of the EC method is to create a high concentration of hydroxide ions ${\left(\text{OH}\right)}^{-}$ in the vicinity of the cathode by chemical reactions (Eqs 4 and 5) (Park et al., 2007).

$$2H_{2}O+2e^{-}\to 2OH^{-}+H_{2}↑$$ (4)

$$2H_{2}O+4e^{-}\to O_{2}+4OH^{-}$$ (5)

The hydroxide ions play an essential role in removing the hardness ions by the reactions, as shown in Eqs 6–8.

$$OH^{-}+HCO_{3}\to CO_{3}^{2-}+H_{2}O$$ (6)

$$Mg^{2+}+2OH^{-}\to Mg{\left(OH\right)}_{2}↓$$ (7)

$$Ca^{2+}+CO_{3}^{2-}\to CaCO_{3}↓$$ (8)

**FIGURE 1:**
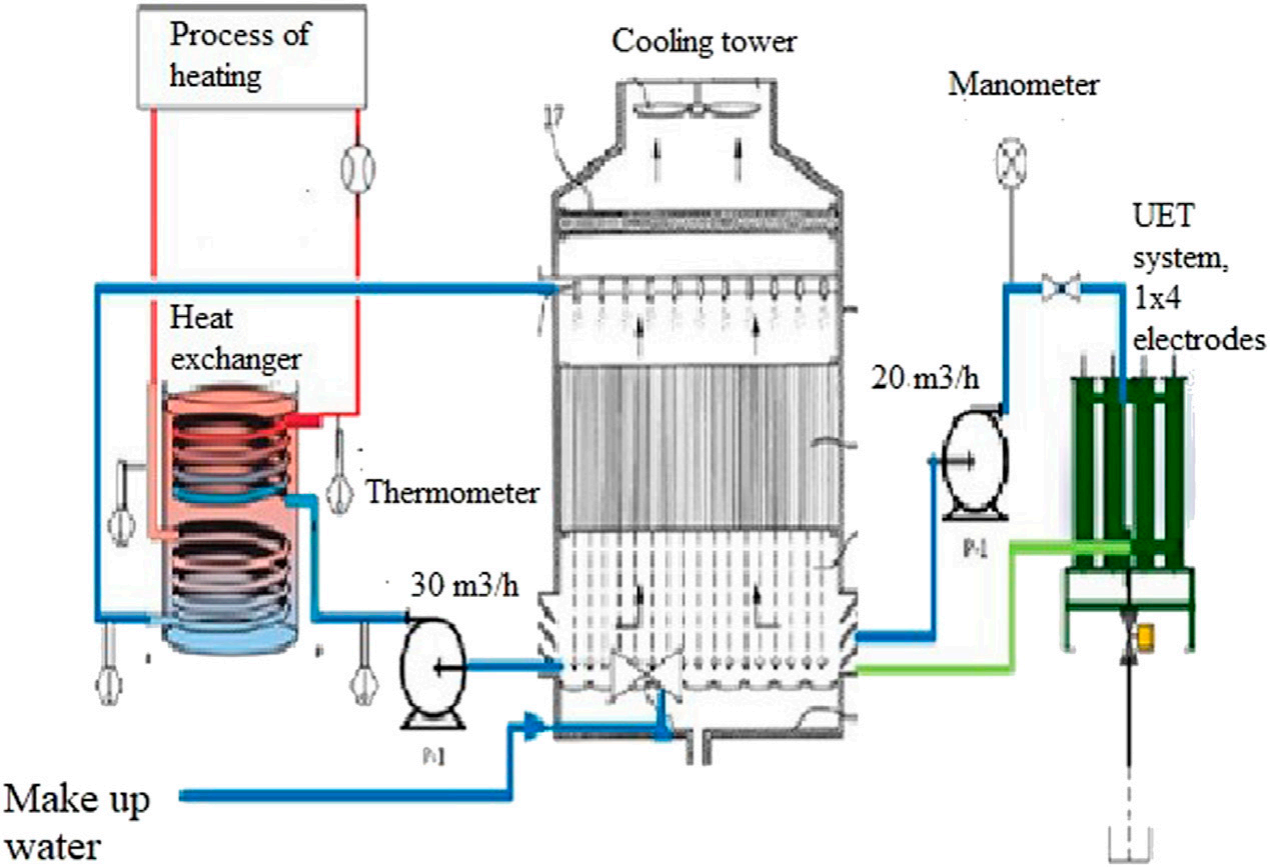
Studied commercial system composed of the cooling tower with basin, heat exchangers, pumps and additional pilot EC reactor (UET System).

The aim of the study was the application of a titanium electrode for the water treatment plant in a steel manufacturing plant to examine the effectiveness of a pilot electrocoagulation process utilizing titanium rod electrodes to remove hard water impurities from the makeup and process water, as shown in Figure 1.

Precious metal oxides used for making coated titanium electrodes are expensive materials. The amount used in practice is, however, minimized by “activating the electrodes” in the preparation process. In the activation process, a few micrometers of the oxide layer are deposited on a metallic support (usually Ti) by the thermal decomposition method. Oxides are seldom used alone in practical applications; usually, they are doped or mixed with less active oxides of higher chemical stability. A DC voltage is applied on the electrodes with periodic polarity reversal to clean the cathode of the hard water scales deposited on it. During the water electrolysis, the film scale deposits [consisting mainly of Ca(OH)_2_, CaCO_3_, and Mg(OH)_2_] form on the cathode surface due to the local increase in pH caused by the evolution of hydrogen (Eq. 4). However, the pH on the anode surface decreases due to the evolution of oxygen (Eq. 5).

## 2 Research methodology

### 2.1 Experimental set-up

The enclosed studied commercial system was in use in a steel manufacturing plant and was composed of the steel manufacturer’s cooling tower with a basin, heat exchangers, pumps, and additional pilot EC reactor (Universal Environmental Technologies, UET system), as shown in Figure 1.

Water was added to the basin of the cooling tower, from where it was pumped to the heat exchanger at a flow rate of 30 m^3^/h.

In the heat exchanger, it was heated to 45°C and then pumped into the cooling tower, where part of the water was condensed and part of it was evaporated.

Water was pumped from the cooling tower basin at a flow rate of 20 m^3^/h to the UET system housing 1 × 4 electrodes, where the minerals were removed and water hardness was reduced. Finally, from the UET system, water was returned to the basin.

The pilot EC reactor (Universal Environmental Technologies, UET system) was housed with four titanium electrodes, each 14 cm × 11.5 cm and spaced 0.5 cm apart. The pilot equipment included an electric power source (alternative DC converter) with variable voltage/amperage. The electric field allows a greater suspension of solids than water purification processes that rely solely on chemicals. Thus, the EC, thanks to the created electric field and greater suspension of solids, is improving the coagulation process. As steel pipes were used in the water cooling process, it is clear that over time, due to oxidation, iron hydroxide was released into the water. The formed iron hydroxides, due to the proximity of the anode and the negative electric charge in the UET, are attracted to each other, creating colloids.

Through a dosing pump, a solution of 20 l/h with a calcium hardness of 500 ppm, obtained by the following reaction with NaHCO_3_ (to increase the alkalinity and pH), was occasionally added to the system as during the time, the electrodes had taken all the hardness present in the water, which had led to the corrosion of the water.

$${CaCl}_{2}+2{NaHCO}_{3}↔{CaCO}_{3}+{CO}_{2}+H_{2}O+2NaCl$$

### 2.2 Measurement methods

Concentrations of Ca^2+^ and Mg^2+^ , as well as total hardness, total iron, and alkalinity, were measured in the water with the Palintest Photometer 7100. Each parameter was also measured in Palintest tablets, supplied, and used, according to the manufacturer’s instructions to establish a standard curve. This photometer was used because it covers a full range of test parameters, enabling effective water quality monitoring.

Conductivity was determined using a conductivity meter (AX400, ABB, Stonehouse, Gloucestershire, UK), and the corrosion potential of the water was determined with a corrosion meter.

In Hasson et al. (2008), the filtered cooling tower water was analyzed to determine the scale ion concentration. The analysis was performed using the APHA (2012) standard methods. The cations and anions were determined using the Unico SQ2800 UV/VIS Spectrophotometer and Dionex ICS-1000 Ion Chromatograph. The water pH was measured using the Fisherbrand™ FE150 pH Meter, and the conductivity was measured by the Horiba LAQUA DS70 conductivity meter.

The scale deposit in the UET plant was examined by X‐ray powder diffractometry (XRD) since this technique is very applicable to soil mineralogy and is one of the most common techniques used to examine the deposition-type soiling inside the pipework. To understand the typology of the deposit inside the system, XRD was utilized as a rapid analytical technique primarily used for phase identification of crystalline materials; the analyzed material is finely ground and homogenized, and the average bulk composition is determined. A cross section of the deposit was analyzed by electron microscopy from where an image of the film was obtained (Figure 6).

## 3 Results

The makeup and process water used for the enclosed studied commercial system, pilot EC reactor (Universal Environmental Technologies system, UET system), initially had the following properties: conductivity 370 μs/cm, pH 7.7, total hardness 250 ppm, and total iron 0.14 ppm.

The studied commercial system, a pilot EC reactor (Universal Environmental Technologies system, UET system), was studied from January to May as shown in Table 1.

**TABLE 1:** Process water analysis after the UET treatment.

		31/12	08/01	18/01	25/01	07/02	17/02	24/02	27/03	05/04	13/04	18/04	24/04	06/05	10/05	17/05
CaH	ppm	175	48	37	25	50	63	75	82	85	90	103	147	14	0.7	1.3
MgH	ppm	75	82	63	75	62	62	57	63	64	60	44	67	90	105	117
TotH	ppm	250	130	100	100	112	125	132	145	149	150	147	214	104	106	117
Malk	ppm	75	83	62	67	73	57	63	60	60	70	55	150	247	500	500
Fe	ppm	0.24	0.07	0.03	0.23	0.3	0.7	1.1	2.8	3	3.5	4.7	3.7	2	0.75	0.5
Cl	ppm	3	8	26	23	27	35	40	47	50	60	440	420	375	375	310
Corrosion	MPY	0	0	0	1.2	1.2	1.2	4	30	25	27	23	7	4.3	3.6	3.4

**FIGURE 2:**
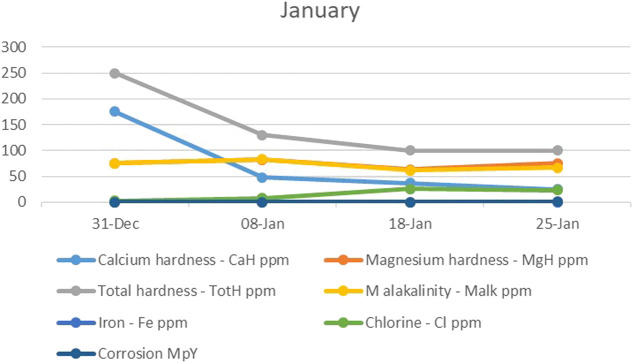
The trend of calcium hardness, magnesium hardness, total hardness, alkalinity, iron, chlorine, and corrosion during the first month.

In the first month of the study (January), the electrodes started to concentrate much more calcium hardness and total hardness than was desirable, while the magnesium hardness did not change greatly. The drop in total hardness led to the alkalinity also dropping, while on the other hand, the corrosion potential increased as the chlorine concentration increased (Figure 2). In addition, with the decreasing concentration of CO_3_/HCO_3_^−^, the hardness removal efficiency and reduction rate of CO_3_/HCO_3_^−^ increased sharply.

**FIGURE 3:**
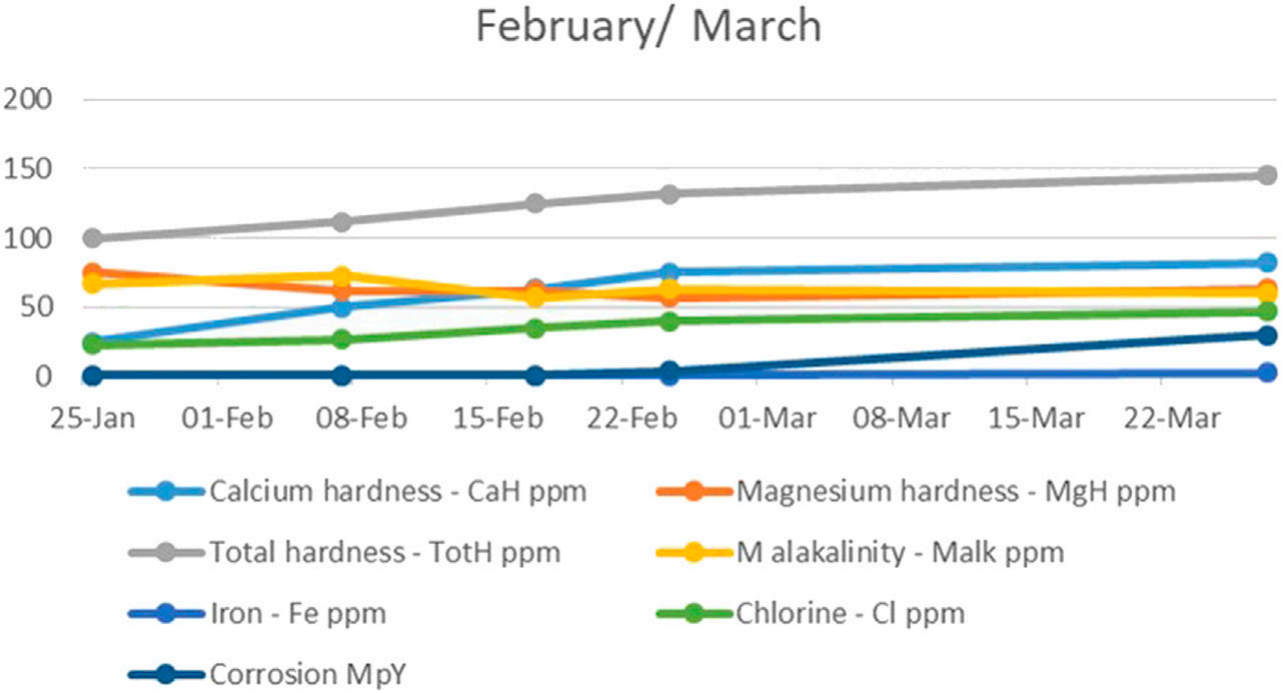
The trend of calcium and magnesium hardness, total hardness, alkalinity, iron, chlorine, and corrosion during the second and third month.

During the second and third months, the level of calcium hardness increased as the number of water circulation cycles through the system was reduced, while the level of magnesium hardness was restored to its initial value. The total alkalinity, however, continued to decrease, while the chlorine level in the water continued to rise. Due to the continuous decrease in alkalinity, corrosion became a serious problem as the extremely high amount of iron grew linearly. The corrosion value went up to 30 MPY in late March (Figure 3). It was assumed this happened because the water did not have the necessary hardness as this was reduced by the electrodes and the number of cycles the water circulated inside the system, which led us to conclude that the set-up was not the best solution for the water in the study.

Also, it became clear that the electrode amperage was very high and that it was not accompanied by water conductivity.

Since the water proved to be corrosive and there was not enough temporary hardness, to make the experimental set-up as close as possible to real conditions, salts in the form of sodium bicarbonate and calcium chloride were added to the system from March.

**FIGURE 4:**
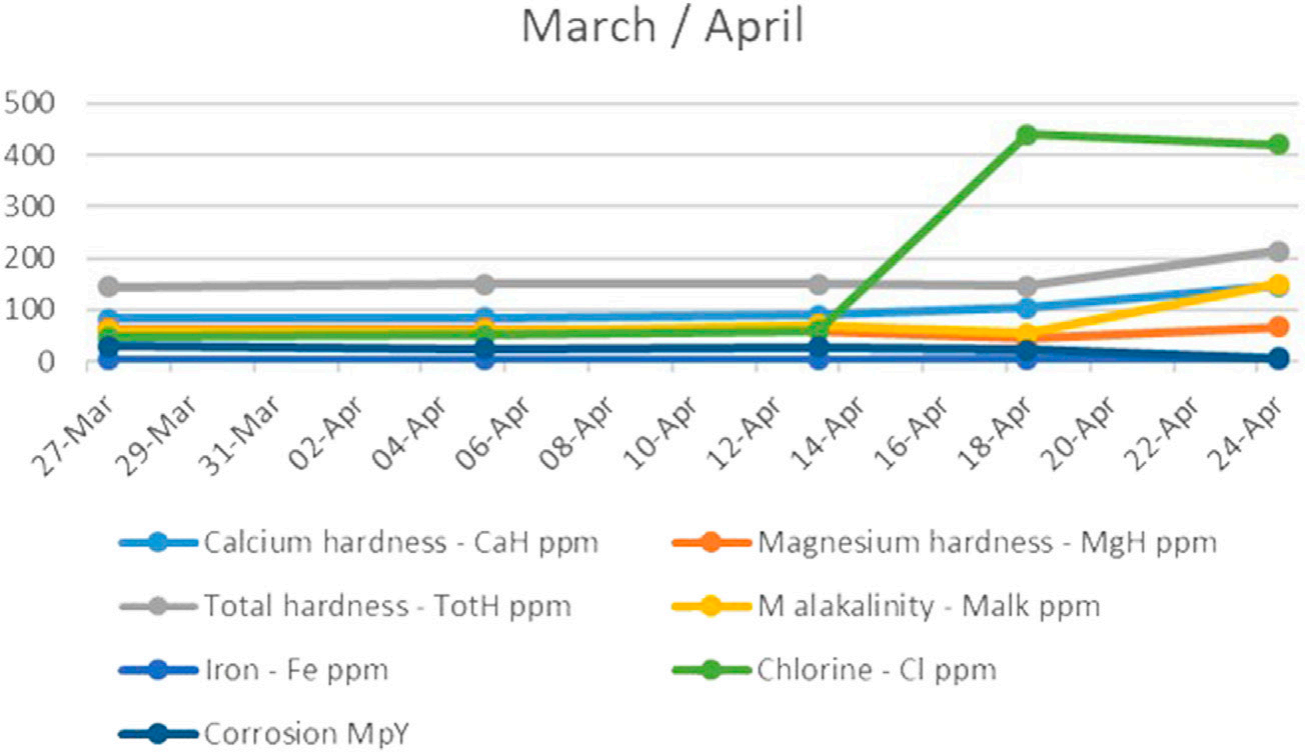
The trend of calcium and magnesium hardness, total hardness, M alkalinity, iron, chlorine, and corrosion during the fourth month.

During the fourth month, the system immediately began to give a positive response, as can be seen in Figure 4, an apparent increase in calcium hardness. However, the magnesium hardness (Figure 4) greatly decreased in the first phase and then returned to its initial, standard, and desired values. This could have been a consequence of the changed pH and alkalinity of the water.

The total alkalinity parameter also increased at that time, along with chlorine levels. As shown in Figure 4, it is interesting to note that the increase in alkalinity had an almost immediate effect on corrosion, independent of the remarkable increase in chlorine that should affect water corrosion. Accordingly, it can be concluded that the corrosion of the water treated in the UET system does not depend on the levels of chlorine in the water.

**FIGURE 5:**
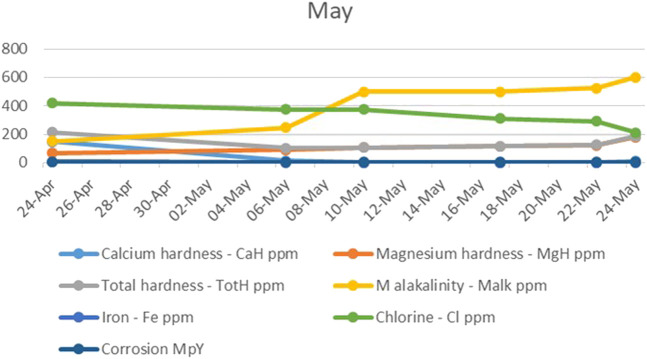
The trend of calcium and magnesium hardness, total hardness, M alkalinity, iron, chlorine, and corrosion during the last month.

In the last month of the study, the problem of scale deposition on the electrodes of the UET system reappeared, in a larger amount than desired, as indicated by the calcium and magnesium hardness levels, as shown in Figure 5. Since the electrodes were set to 12 A, a logical conclusion was that the strength of the electric current potentially contributed to the increased calcium deposition. The electrodes were then manually set to 6 A, to which the system responded well, allowing calcium to be concentrated to the desired values.

The magnesium hardness maintained a steady, constant increase, with a sharp upward increase that accompanied the decrease in electric current.

The total alkalinity also continued to increase in the last month, which could be explained by the fact that the occurrence of strong electromigration, caused by the increase of current, accelerated the migration of HCO_3_^−^ toward the anode, and it then resulted in the decrease of chemical reaction efficiency, while chlorine, iron, and corrosion levels continued to decrease.

Once again, it can be concluded that the corrosion of water treated in the UET system was not directly dependent on the chlorine levels it contained.

## 4 Discussion

With the decreasing concentration of CO_3_/HCO_3_^−^, the hardness removal efficiency and reduction rate of CO_3_/HCO_3_ increased sharply, which was consistent with the previous conclusion (Jin et al., 2019) that alkalinity was the key factor that determines the maximum treatment capacity of scale amounts. Effectively, this means a prolonged electrolysis time could not increase the total amount of deposited scales (Nishida et al., 2009).

The current input, as well, was involved as it is the key parameter determining the concentration of the dosed coagulant in the treated process water. In most published papers, variation in the current input is accompanied by a variation in the current density since the active surface area of the electrode is almost always kept constant (Pavel and Duarte, 2017). Most studies analyzed the current density instead of the current input.

The current density controls the rate of coagulant production, adjusts bubble production, and, hence, affects the growth of flocculation (Mollah et al., 2001; Hafez et al., 2018) which showed that with the increased current density, removal of the total hardness also increased.

Mollah et al. (2001) stated that Al, Fe, and Zn electrodes, under optimal conditions, produced efficient removal of the total hardness, calcium hardness, and magnesium hardness in the necessary, satisfactory range.

In contrast, in the enclosed studied commercial system, the system, despite depositing a certain amount of hardness inside the reactor, did not prevent deposits in the pipes. Notably, the fact that the alkalinity reduction rate gradually became higher than the hardness removal efficiency indicated other pathways had decreased the alkalinity, in addition to the decrease in the formation of CaCO_3_ on the electrodes, which was consistent with the previous studies (Zaslavschi et al., 2013).

Zhang et al. (2020) have shown that the fouling rate increased rapidly on the base of the scale layer and then gradually decreased. This is in line with the experimental data (Zhang et al., 2020) because the generation of OH^−^ ions was gradually shifted from the external layers to the internal layers of the electrodes to make a large amount of OH^−^ ions diffuse toward the solution outside the scale layer. Once in the solution, those ions reacted with Ca^2+^ and Mg^2+^ ions near the external layers of the electrodes.

In addition, the inner electrode distance with the constant current density was also considered in the current study. The short distance between the electrodes (0.5 cm) resulted in a low flow of the solution from one electrode to the next, this likely negatively affected the scale ion removal efficiency. A further increase in the distance between the electrodes, up to 1 cm, should improve the efficiency of scale ion removal, but further increasing the distance between the electrodes reduces the efficiency. This is mainly due to the weak interactions between the generated flocs and the scale molecules (Mollah et al., 2001). In the enclosed studied commercial system, the 0.5 cm distance between electrodes resulted in a greater removal of total hardness, so it can be concluded that it was not a problem with the inner distance between the electrodes. The problem was that the current input was very high for the desirable removal of the impurities. This produced water that had corrosive properties, which meant that equipment inside the plant was harmed because of the deleterious water quality.

The makeup water circulating through the system was colored red due to corrosion of the steel pipes. The factors that have a significant impact on the occurrence of corrosion within the system are pH, temperature, dissolved oxygen, carbon dioxide, suspended solids, etc. Steel is often used in such systems primarily due to its mechanical properties, high thermal conductivity, and low cost. In essence, steel at high temperatures, under the conditions of alkaline reduction, forms a thin protective layer of adhering magnetite oxide (Fe_3_O_4_) on the surface that protects the base metal from corrosion by the aquatic environment.

The red color is due to the formation of Fe^2+^ ions, which are the first products of corrosion and which easily oxidize under normal conditions to Fe^3+^ (Flynn, 2009).

**FIGURE 6:**
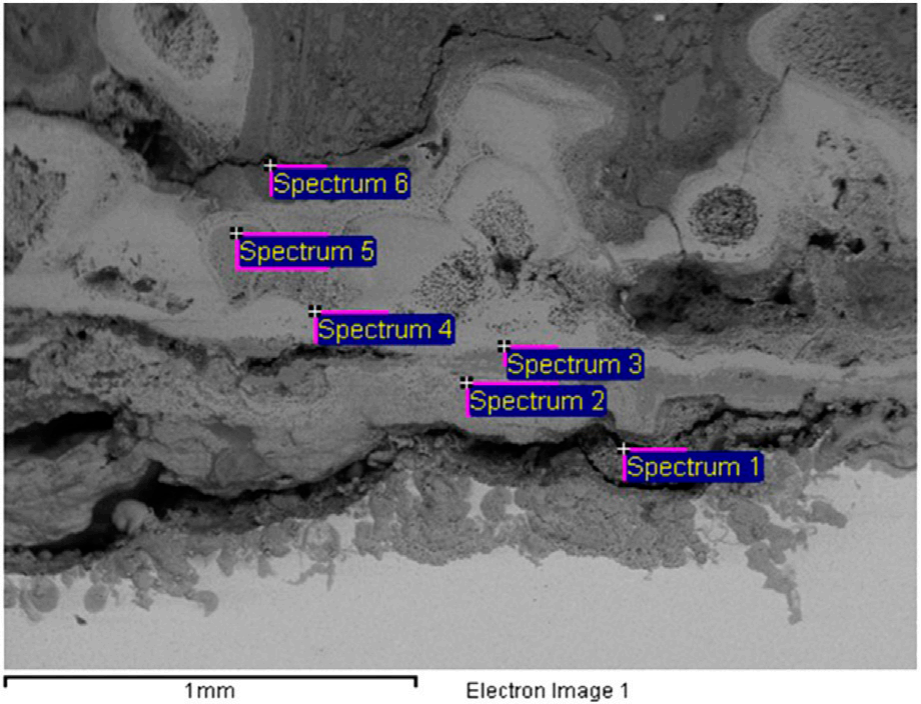
Image of the cross-section of the deposition inside the pipe.

Moreover, at the end of the pilot study with the UET system, after dismantling the plant, a thick layer of sediment on the surface of the pipes was discovered, and its composition was determined. The scale deposit was rich in calcium, iron, and chloride (Figure 6; Table 2), which explained the relatively low concentrations of chlorides present in the water and the corrosion that had occurred.

**TABLE 2:** Elemental composition (in %) of the deposit inside the pipes.

Spectrum	C	O	Al	Si	S	Cl	Ca	Mn	Fe	Cu	Br	Total
Spectrum 1	14.90	33.94	0.78	0.44		2.19	0.28		46.79	0.68		100.00
Spectrum 2	8.66	31.73		0.73		5.61			53.26			100.00
Spectrum 3	9.88	37.04		0.42	0.30			0.43	51.92			100.00
Spectrum 4	3.79	30.83		0.41					64.97			100.00
Spectrum 5	10.12	36.16		0.54		0.32	0.86		51.99			100.00
Spectrum 6	28.35	37.17		3.90		0.40	16.90		6.95		6.34	100.00

Different points of the pipes were analyzed, in plan and section, always giving similar compositions.

This composition is showing once more that everything that was not removed with the UET system was deposited inside the plant.

## 5 Conclusion

At the end of the study, the closed water system was dismantled to reveal a thick layer of sediment on the pipe surfaces. Examination of the deposit with an X‐ray powder diffractometer confirmed all assumptions made during the study. The system was unable to deposit the necessary compounds and elements from the water, in desired quantities such as to bring the water to a “natural” balance. The system, despite depositing a certain amount of scales inside the reactor, did not prevent deposits in the pipes. This effect is explained by the fact that the system itself was unable to perform self-regulation. The conductivity-based control only served to ensure that the voltage remained constant. In our case, the water with low hardness and the system with a constant voltage meant that the system removed a much larger amount of hardness than needed, causing the water to become corrosive and the corrosion itself to increase inside the plant.

However, this kind of problem could be resolved with the upgradation of the UET system. It is clear that the system is capable of removing scale ions from the water, but to be able to maintain a necessary equilibrium of the water, a self-regulation system should be installed. This self-regulation system should be set to follow the mentioned and analyzed parameters (calcium and magnesium hardness, total hardness, iron, chlorine, M alkalinity, and corrosion) and their maximum allowed limits. In case it goes beyond the limits, which were set up, the system would be able to recognize that the limits were crossed and would be able to regulate the amperage/voltage inside of the system. It is proven that with the upgradation of the system with self-regulation equipment, the necessary performance and desirable water quality inside a closed system can be obtained.

## Data Availability

The original contributions presented in the study are included in the article/Supplementary Material; further inquiries can be directed to the corresponding author.
